# BORC complex specific components and Kinesin-1 mediate autophagy evasion by the autophagy-resistant *Mycobacterium tuberculosis* Beijing strain

**DOI:** 10.1038/s41598-023-28983-5

**Published:** 2023-01-30

**Authors:** Janpen Tunganuntarat, Phongthon Kanjanasirirat, Tanawadee Khumpanied, Salisa Benjaskulluecha, Benjawan Wongprom, Tanapat Palaga, Tegar Adriansyah Putra Siregar, Suparerk Borwornpinyo, Angkana Chaiprasert, Prasit Palittapongarnpim, Marisa Ponpuak

**Affiliations:** 1 Department of Microbiology, Faculty of Science, Mahidol University, Rama VI Road, Bangkok, 10400 Thailand; 2 Faculty of Science, Excellent Center for Drug Discovery, Mahidol University, Bangkok, Thailand; 3 Medical Microbiology, Interdisciplinary Program, Graduate School, Chulalongkorn University, Bangkok, Thailand; 4 Department of Microbiology, Faculty of Science, Chulalongkorn University, Bangkok, Thailand; 5 Department of Microbiology, Faculty of Medicine, University of Muhammadiyah Sumatera Utara, Medan, Indonesia; 6 Department of Biotechnology, Faculty of Science, Mahidol University, Bangkok, Thailand; 7 Drug-Resistance Tuberculosis Research Fund, Siriraj Foundation, Faculty of Medicine Siriraj Hospital, Mahidol University, Bangkok, Thailand; 8 Office of Research and Development, Faculty of Medicine Siriraj Hospital, Mahidol University, Bangkok, Thailand; 9 National Center for Genetic Engineering and Biotechnology, National Science and Technology Development Agency, Pratumthani, Thailand; 10 Department of Microbiology, Faculty of Science, Pornchai Matangkasombut Center for Microbial Genomics, Mahidol University, Bangkok, Thailand

**Keywords:** Cell biology, Microbiology

## Abstract

Autophagy induction by starvation has been shown to enhance lysosomal delivery to mycobacterial phagosomes, resulting in the restriction of the *Mycobacterium tuberculosis* reference strain H37Rv. In contrast to H37Rv, our previous study showed that strains belonging to the notorious *M. tuberculosis* Beijing genotype could evade autophagic elimination. Our recent RNA-Seq analysis also discovered that the autophagy-resistant *M. tuberculosis* Beijing strain (BJN) evaded autophagic control by upregulating the expression of *Kxd1*, a BORC complex component, and *Plekhm2*, both of which function in lysosome positioning towards the cell periphery in host macrophages, thereby suppressing enhanced lysosomal delivery to its phagosome and sparing the BJN from elimination as a result. In this work, we further characterised the other specific components of the BORC complex, BORC5-8, and Kinesin proteins in autophagy resistance by the BJN. Depletion of BORCS5-8 and Kinesin-1, but not Kinesin-3, reverted autophagy avoidance by the BJN, resulting in increased lysosomal delivery to the BJN phagosomes. In addition, the augmented lysosome relocation towards the perinuclear region could now be observed in the BJN-infected host cells depleted in BORCS5-8 and Kinesin-1 expressions. Taken together, the data uncovered new roles for BORCS5-8 and Kinesin-1 in autophagy evasion by the BJN.

## Introduction

Tuberculosis (TB) caused by the bacterium *Mycobacterium tuberculosis* ranks as the thirteenth major cause of death worldwide^1^. TB most commonly affects the lungs and can spread from an infected person to others through the air. When people inhale the infectious droplet nuclei containing the mycobacteria released from active TB patients through coughing or sneezing, they become infected^2^. Although antibiotic treatment is effective against the drug-susceptible *M. tuberculosis* strains, infections caused by drug-resistant and multi-drug-resistant *M. tuberculosis* are on the rise^3^. In addition, long-period drug treatment for the drug-susceptible *M. tuberculosis* strains can result in the accumulation of drug-resistant alleles by mutation of the *M. tuberculosis* antibiotic target genes^4^. Due to the drug resistance problem mentioned above, modulation of specific host pathways by host-directed therapeutics is considered a promising new approach for TB treatment combination therapy.

Autophagy is a conserved cellular process important for maintaining cellular homeostatic balance in the human body^5^. Under normal conditions, this mechanism is present at a basal level in our cells, but stressors such as nutrient deprivation can increase its activation^6^. Autophagy dysregulation has been linked to a number of human disorders such as cancer, metabolic dysfunction, neurodegeneration and inflammatory diseases^7^. Autophagy not only facilitates the elimination of deteriorated cellular components to prevent cellular dysfunction and disease development but also contributes to the eradication of intracellular pathogens including *M. tuberculosis*^8^. During autophagy, the substrates are engulfed by the double-membrane autophagosomes followed by delivery to the acidic lysosomes located in the perinuclear region, which contain lysosomal hydrolases for eliminating the sequestered contents^9^. Peripheral lysosomes also relocate towards the perinuclear region for fusion with autophagosomes during nutrient starvation^10^. Our work as well as that of others showed that starvation-induced autophagy in host macrophages resulted in the restriction of the *M. tuberculosis* reference strain H37Rv^11–18^. In contrast to H37Rv, we recently showed that strains belonging to the *M. tuberculosis* Beijing genotype can resist autophagic restriction^11,19,20^. The escape of the Beijing strains from starvation-induced autophagic elimination was not due to the inhibition of the general host cell autophagic flux or autophagy-mediated acidification of their phagosomes^11^. However, the Beijing strains evade autophagy-mediated restriction by blocking the xenophagic flux as demonstrated by the significant decrease in the lysosomal delivery into their phagosomes during starvation-induced autophagy^11^. We discovered that while lysosomes were delivered to the H37Rv phagosomes upon autophagy induction by starvation dependent upon Beclin-1, lysosomal delivery to phagosomes of the autophagy-resistant Beijing strains was inhibited^11^. Of note, the *M. tuberculosis* Beijing family has been shown to be associated with drug resistance^21–25^ and hyper virulence, including increased mycobacterial survival inside host macrophages, fatality rates in animal models and the number of acid-fast bacilli in TB patient sputum^23,26–30^. In addition, by using RNA-Seq analysis we recently demonstrated that the autophagy-resistant Beijing strain (BJN) can evade autophagic restriction by upregulating the expression of *Kxd1*, a member of the BORC complex, and *Plekhm2*, both of which are involved in lysosome positioning towards the cell periphery in host macrophages, thus resulting in the relocation of lysosomes away from the perinuclear region, decrease in lysosome fusion with the BJN phagosomes, and sparing of the BJN from elimination^19^.

The BORC complex consists of eight proteins: BLOC1S1 (BLOS1 or BORCS1), BLOS2 (BLOS2 or BORCS2), Snapin (BORCS3), Kxd1 (BORCS4), Myrlysin (BORCS5), Lyspersin (BORCS6), Diaskedin (BORCS7), and MEF2BNB (BORCS8)^31,32^. BORCS1, BORCS2 and Snapin were also reported to be part of another complex called BLOC-1, which is involved in the biogenesis of lysosome-related organelles^31,32^. It was shown that the knockout of BLOC-1 subunits does not affect lysosome positioning, while the knockout and knockdown of BORC-specific subunits interfere with the distribution of lysosomes, resulting in the accumulation of lysosomes in the perinuclear region^31^. It was also found that the BORC complex can move the lysosomes towards the cell periphery by 2 slightly different mechanisms. First, the BORC complex recruits Arl8, which in turn recruits Plekhm2, which then interacts with Kinesin-1 to transport lysosomes on the microtubules enriched in acetylated α-tubulin towards the cell periphery^32^. Alternatively, the BORC-Alr8 complex can directly recruit Kinesin-3 to traffic lysosomes on the microtubules enriched in tyrosinated α-tubulin towards the cell periphery^32^. Our previous work showed that *Kxd1* and *Pleckhm2* are necessary for the BJN’s evasion from autophagic restriction^19^, but whether other BORC complex components, Kinesin-1 and/or Kinesin-3 are involved in this process remains to be determined.

In this study, we examined the roles of other BORC complex components, Kinesin-1 and Kinesin-3 in autophagy resistance by the BJN. We found that BORCS5-8 and Kinesin-1, but not Kinesin-3, are important for autophagy evasion by the BJN. Thus, our findings further emphasize the important roles of the BORC complex components and Kinesin-1 in autophagy evasion by the BJN and provide a drug target to counteract autophagy resistance by the *M. tuberculosis* Beijing strain.

## Results

### BORCS5-8 and Kinesin-1, but not Kinesin-3, are necessary for the evasion of autophagic restriction by the BJN

Our previous work utilising RNA-Seq followed by phenotypic analysis showed that, unlike the autophagy-sensitive *M. tuberculosis* reference strain H37Rv, the autophagy-resistant *M. tuberculosis* BJN strain induces the expression of *Kxd1*, a component of the BORC complex, and *Plekhm2*, both of which function in lysosome positioning towards the cell periphery, in autophagy-induced host macrophages, resulting in the decreased colocalisation of lysosomes with the BJN phagosomes and resistance of the BJN to autophagic elimination^19^. In this study, we examined the roles of other BORC complex-specific components, BORCS5-8, in the autophagy evasion by the BJN. Moreover, we tested the roles of motor proteins in the autophagy resistance by the BJN as well since the BORC complex can move lysosomes towards the cell periphery using Kinesin-1 or Kinesin-3^32^.

**Figure 1:**
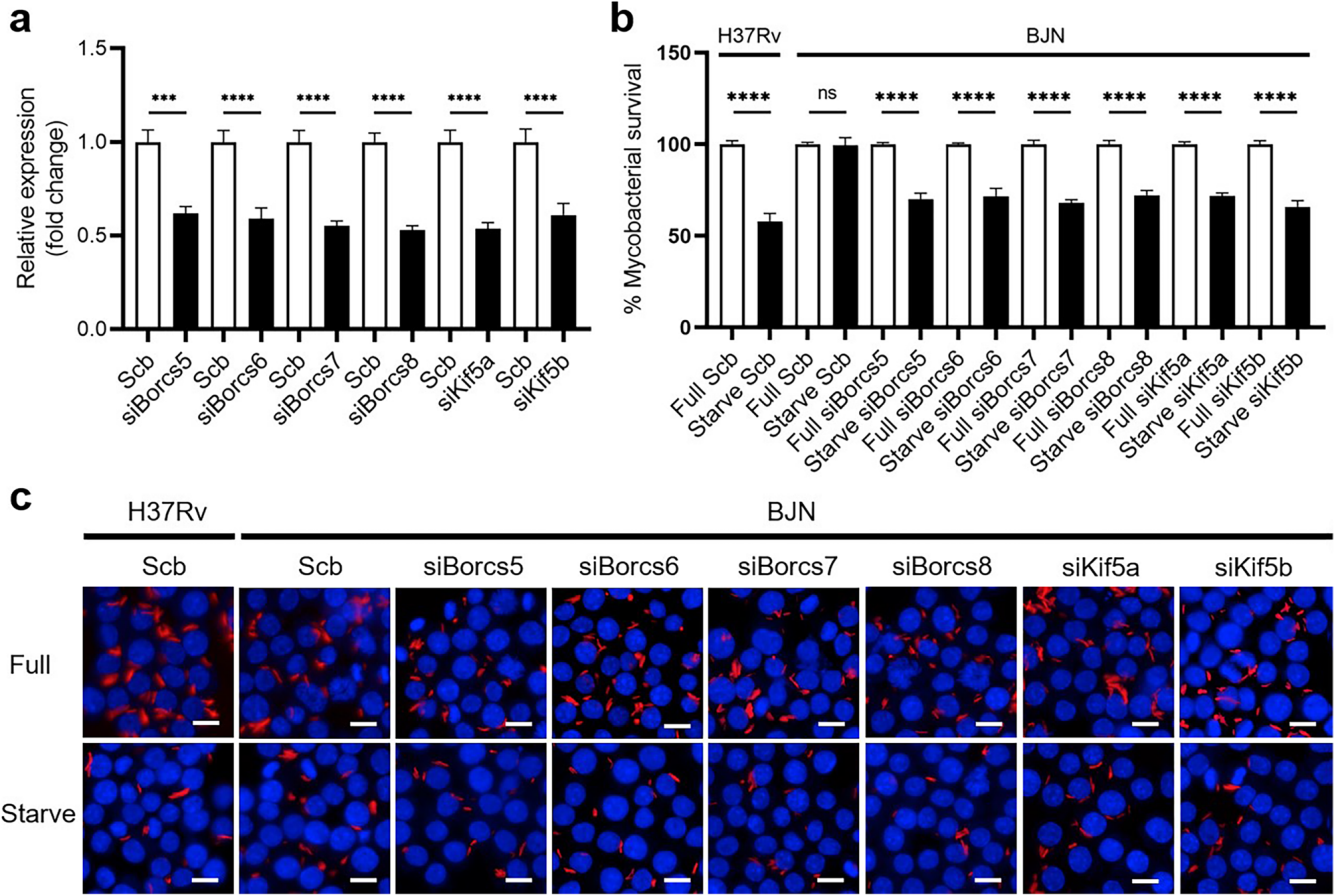
BORCS5-8 and Kinesin-1 are important for the evasion of autophagic restriction by the BJN in RAW264.7 macrophages. (**a**) RAW264.7 macrophages were transfected with siRNAs against *Borcs5*, *Borcs6*, *Borcs7*, *Borcs8*, *Kif5A*, and *Kif5B* or scrambled control siRNAs by nucleofection for 48 h. Knockdown efficiency was determined by qRT-PCR. Expression levels of the target genes were normalised to that of the housekeeping gene, *Gapdh*. Data are means ± SEM from at least three independent experiments; ***p < 0.001 and ****p < 0.0001, relative to the scrambled siRNA control set to 1.0 determined by one-way ANOVA with Tukey’s multiple comparison test. (**b,c**) RAW264.7 macrophages deficient in BORC5-8 and Kinesin-1 were infected with mCherry-expressing H37Rv or BJN (MOI = 10) for 1 h followed by autophagy induction by starvation for 4 h. The number of intracellular mycobacteria per cell was then determined by high-content imaging, and percent of mycobacterial survival was calculated and compared (**b**). Data are means ± SEM from at least three independent experiments; *ns* non-significant and ****p < 0.0001, all relative to the full control set of 100% determined by one-way ANOVA with Tukey’s multiple comparison test. Representative images are displayed in (**c**). Bar 10 µm.

To determine this, the expression of *Borcs5-8*, *Kif5a* and *Kif5b* (encoding for Kinesin-1 in monocytes/macrophages), and *Kif1b* (encoding for Kinesin-3 in monocytes/macrophages) were first suppressed by using siRNA knockdown technology in RAW264.7 macrophages. Successful knockdown of the aforementioned genes was confirmed by qRT-PCR (Fig. 1a, Supplementary Fig. S1). The mycobacterial survival assay based on high-content image analysis was then performed upon autophagy induction in RAW264.7 macrophages, as previously described^19,20^. In agreement with our prior findings^11,19,20^, autophagy induction by starvation of host macrophages restricted H37Rv, while the BJN resisted such elimination in scrambled siRNA-transfected control cells (Fig. 1b,c, Supplementary Fig. S1). Interestingly, the BJN could now be restricted by starvation-induced autophagy upon depletion of *Borcs5-8*, *Kif5a* and *Kif5b*, but not *Kif1b*, from RAW264.7 macrophages (Fig. 1b,c, Supplementary Fig. S1). These findings revealed that BORCS5-8 and Kinesin-1, but not Kinesin-3, are required for the evasion of autophagic restriction by the BJN.

### BORCS5-8 and Kinesin-1 are important for the BJN to evade lysosomal delivery during starvation-induced autophagy

**Figure 2:**
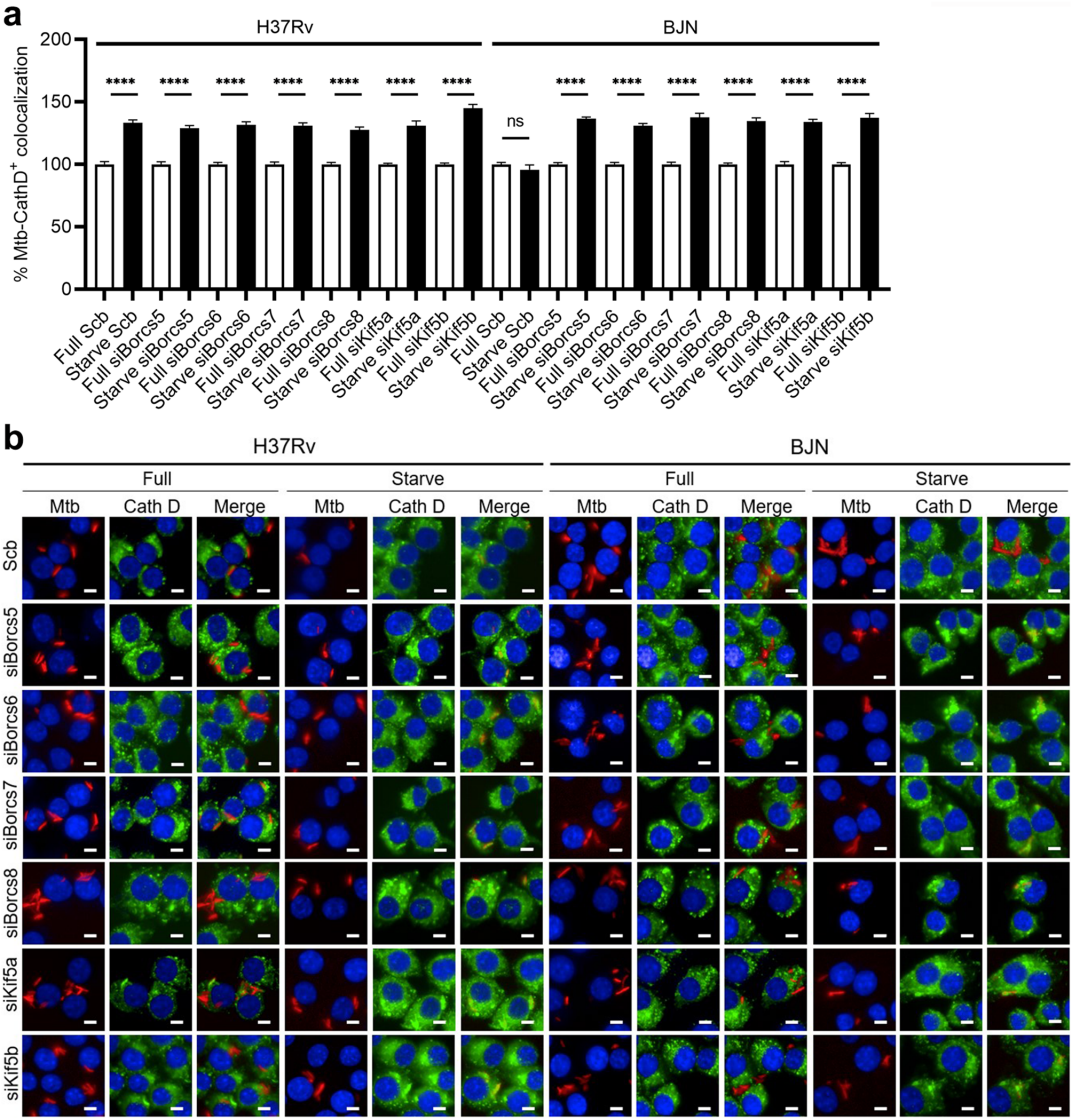
BORCS5-8 and Kinesin-1 impair lysosomal delivery to the BJN phagosomes in RAW264.7 macrophages. (**a,b**) RAW264.7 macrophages depleted in BORCS5-8 and Kinesin-1 expression were infected with the mCherry-expressing H37Rv or BJN for 15 min and chased for 1 h. Autophagy was then induced by starvation for 2 h. Cells were fixed and stained for lysosomes using an anti-Cathepsin D antibody followed by nuclear labelling with Hoechst. Percent mycobacteria-Cathepsin D colocalisation was then analysed by high-content image analysis. Data are means ± SEM from at least three independent experiments; *ns* non-significant and ****p < 0.0001, all relative to the full control set of 100% determined by one-way ANOVA with Tukey’s multiple comparison test (**a**). Representative images are displayed in (**b**). Bar 5 µm.

To further evaluate whether the reverted resistance phenotype of the BJN to starvation-induced autophagy observed above was due to an increase in lysosomal delivery to the BJN phagosomes, we examined the effects of *Borcs5-8*, *Kif5a*, and *Kif5b* depletion on the colocalisation of the mycobacteria with Cathepsin D, used as a marker for lysosomes, in RAW264.7 macrophages. Consistent with our previous results^11,19,20^, the increase in colocalisation of Cathepsin D with H37Rv phagosomes was observed in scrambled siRNA-transfected H37Rv-infected RAW264.7 macrophages induced to undergo autophagy by starvation, but such an effect was not observable in the BJN-infected control cells (Fig. 2a,b). Furthermore, the enhanced colocalisation of Cathepsin D with the BJN phagosomes could now be observed during autophagy induction by starvation upon reducing the expression of *Borcs5-8*, *Kif5a* and *Kif5b* from RAW264.7 macrophages (Fig. 2a,b). Of note, upon starvation we detected no difference in the enhanced Cathepsin D levels in H37Rv- or BJN-infected RAW264.7 macrophages transfected with scrambled control siRNAs or siRNAs against *Borcs5-8*, *Kif5a*, and *Kif5b* (Supplementary Fig. S2). These findings demonstrated that BORCS5-8 and Kinesin-1 play critical roles in limiting lysosomal delivery to the BJN phagosomes in response to autophagy activation by the starvation of host cells.

### BORCS5-8 and Kinesin-1 mediate enhanced lysosome positioning towards the cell periphery by the BJN

**Figure 3:**
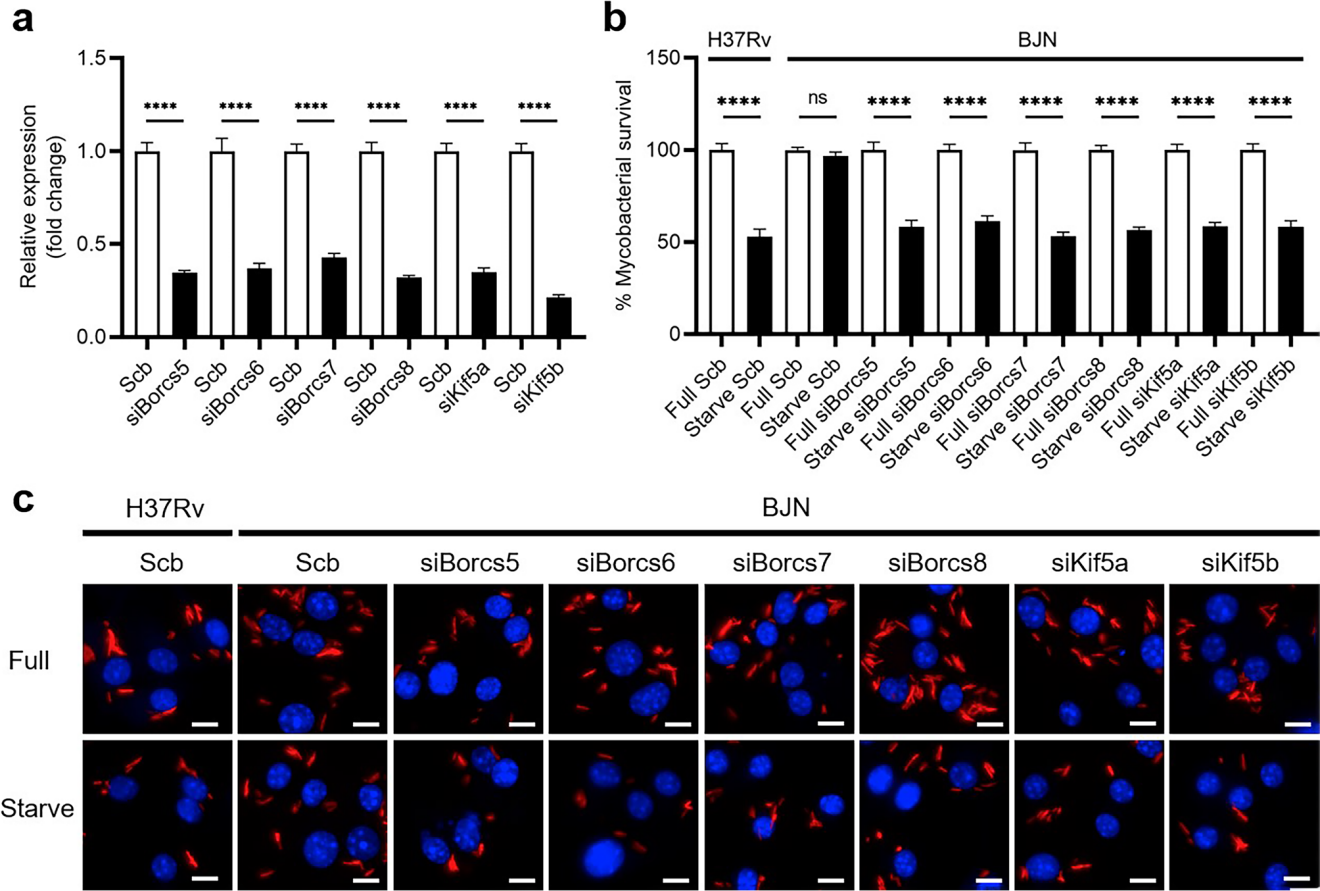
Depletion of BORCS5-6 and Kinesin-1 reverts the BJN’s ability to resist autophagic elimination in BMDMs. (**a**) BMDMs were transfected with siRNAs, as in Fig. 1. Successful knockdown was determined by qRT-PCR. Expression levels of the target genes were normalised to that of the housekeeping gene, *Gapdh*. Data are means ± SEM from at least three independent experiments; ****p < 0.0001, relative to the scrambled siRNA control set to 1.0 determined by one-way ANOVA with Tukey’s multiple comparison test. (**b,c**) BMDMs with decreased expression of BORCS5-8 and Kinesin-1 were infected with the mCherry-expressing H37Rv or BJN and induced to undergo autophagy by starvation, as in Fig. 1. High-content imaging was then used to assess the number of intracellular mycobacteria per cell. Percent mycobacterial survival was calculated and compared (**b**). Data are means ± SEM from at least three independent experiments; ns, non-significant and ****p < 0.0001, all relative to the full control set of 100% determined by one-way ANOVA with Tukey’s multiple comparison test. Representative images are displayed in (**c**). Bar 10 µm.

To investigate whether increased lysosomal delivery to the BJN phagosomes and the reverted resistance phenotype of the BJN to starvation-induced autophagy observed above upon reduced expression of BORCS5-8 and Kinesin-1 were the results of the decrease in lysosome positioning towards the cell periphery, we analysed lysosome position in the BJN-infected BMDMs. Notably, the size of RAW264.7 macrophages is too small to conduct a lysosome positioning analysis^19^. To determine this, we first confirmed the important roles of BORCS5-8 and Kinesin-1 in autophagy evasion by the BJN in BMDMs. *Borcs5-8*, *Kif5a* and *Kif5b* expressions were knocked down in the BMDMs by using siRNA knockdown technology. The qRT-PCR results showed the successful depletion of the aforementioned genes in the BMDMs (Fig. 3a). In agreement with our previous data^19^, a decrease in H37Rv intracellular survival was observed in the scrambled siRNA-transfected BMDMs induced to undergo autophagy by starvation, but the effect was not observable in the BJN-infected control cells (Fig. 3b,c). Consistent with the results observed in RAW264.7 macrophages, the BJN could now be restricted during starvation-induced autophagy in BMDMs upon BORCS5-8 and Kinesin-1 depletion (Fig. 3b,c). These data confirmed the necessary roles of BORCS5-8 and Kinesin-1 in the evasion of autophagic restriction by the BJN in BMDMs.

**Figure 4:**
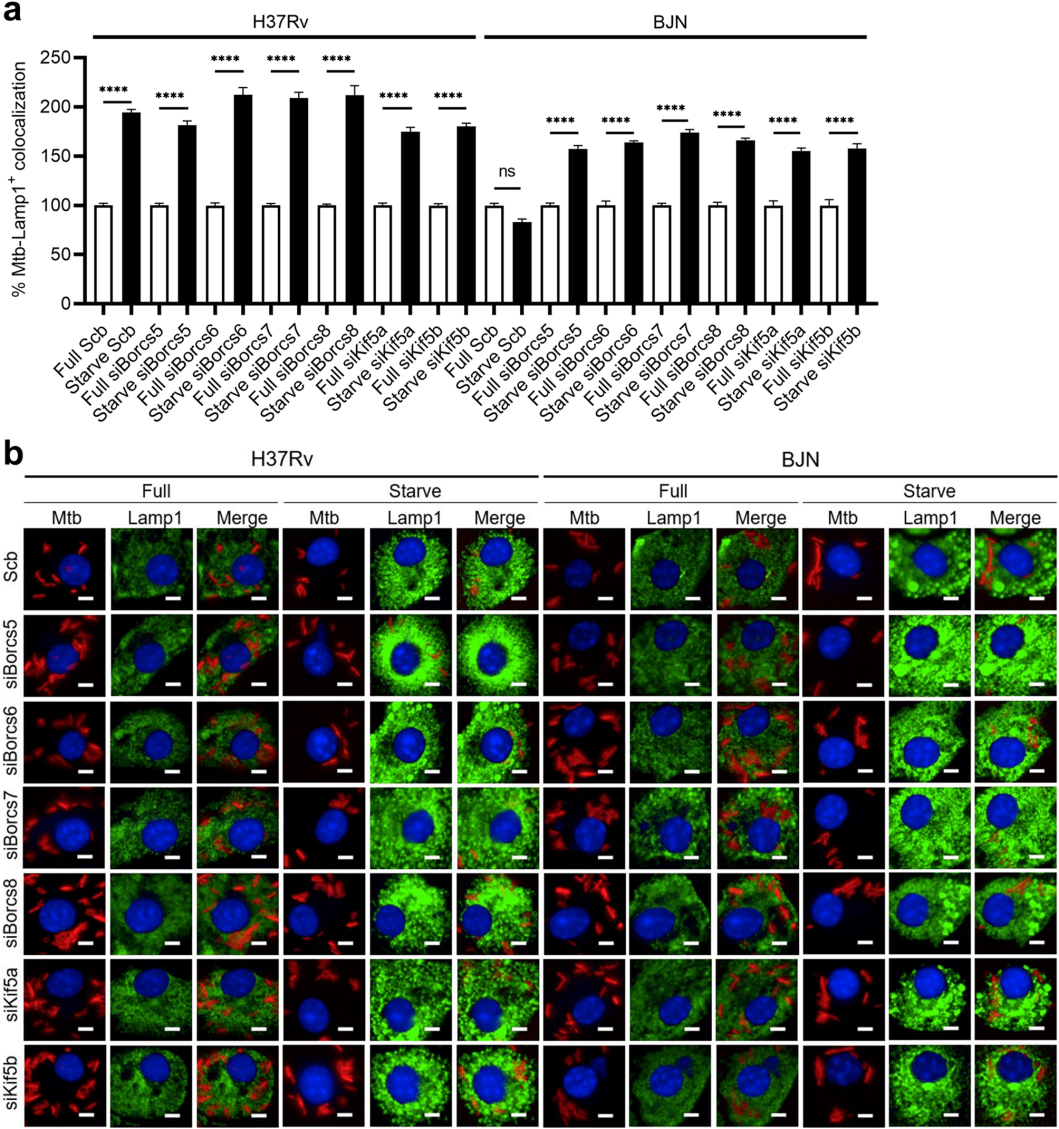
BORCS5-8 and Kinesin-1 suppress lysosome delivery to the BJN phagosomes in BMDMs. (**a,b**) BMDMs deficient in BORCS5-8 and Kinesin-1 expression were infected with the mCherry-expressing H37Rv or BJN and induced to undergo autophagy by starvation, as in Fig. 2. Cells were fixed and stained for lysosomes using an anti-Lamp1 antibody followed by nuclear staining with Hoechst. Percent mycobacteria-Lamp1 colocalisation was then analysed by high-content image analysis. Data are means ± SEM from at least three independent experiments; ns, non-significant and ****p < 0.0001, all relative to the full control set of 100% determined by one-way ANOVA with Tukey’s multiple comparison test (**a**). Representative images are displayed in (**b**). Bar 5 µm.

Next, we evaluated the roles of BORCS5-8 and Kinesin-1 in the reduced lysosomal delivery to the BJN phagosomes in BMDMs. To determine this, BMDMs depleted in BORCS5-8 and Kinesin-1 expressions were infected with H37Rv or BJN and induced to undergo autophagy by starvation. Colocalisation of the mycobacteria with lysosomes was analysed by high-content imaging using staining lysosomes with anti-Lamp1 antibodies. Consistent with our previous work^19^, the increase in H37Rv-Lamp1 colocalisation, but not that of BJN, was observed in scrambled siRNA-transfected BMDMs upon autophagy induction by starvation (Fig. 4a,b). In agreement with our results in RAW264.7 macrophages, upon depletion of BORCS5-8 and Kinesin-1, the enhanced colocalisation of Lamp1 with the BJN phagosomes could now be noted (Fig. 4a,b). Importantly, when H37Rv- or BJN-infected BMDMs transfected with scrambled control siRNAs or siRNAs against *Borcs5-8*, *Kif5a*, and *Kif5b* were induced to undergo autophagy by starvation, no dissimilarity in the increased Lamp1 levels was seen as shown in Supplementary Fig. S3. Therefore, these findings supported the vital roles of BORCS5-8 and Kinesin-1 in decreased lysosomal delivery to the BJN phagosomes during starvation-induced autophagy in BMDMs.

**Figure 5:**
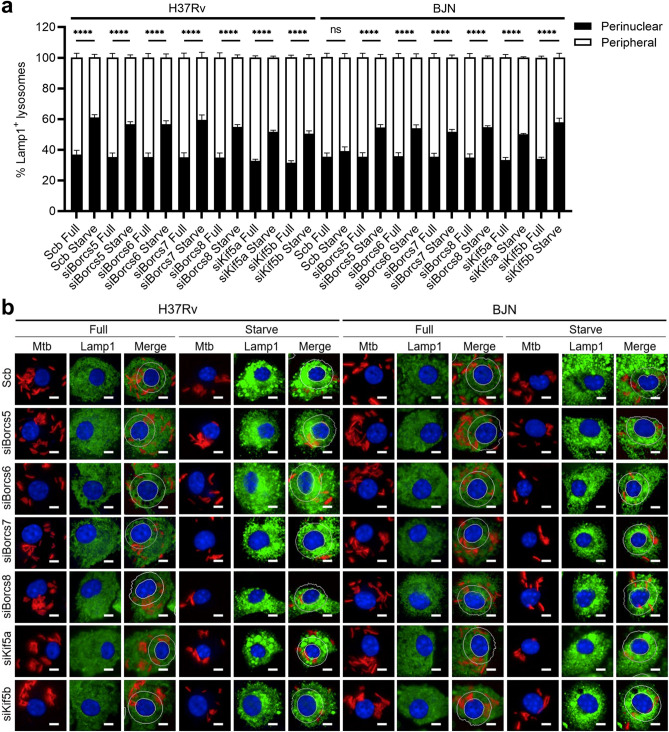
BORCS5-8 and Kinesin-1 dampen lysosome relocation to the perinuclear area in the BJN-infected BMDMs. (**a,b**) BORCS5-8- and Kinesin-1-depleted BMDMs were infected with the mCherry-expressing H37Rv or BJN for 15 min and chased for 1 h, as in Fig. 4. Autophagy was then induced by starvation for 24 h. Cells were stained with anti-Lamp1 antibody and Hoechst and then processed for high-content image analysis. The number of Lamp1^+^ lysosomes in each cytoplasmic subregion of the mycobacteria-infected BMDMs was quantified. The percentage of perinuclear Lamp1^+^ lysosomes (0–4 µm distance from the nucleus) and peripheral Lamp1^+^ lysosomes (4 µm from the nucleus and cell boundary) were then calculated and compared. Data are means ± SEM from at least three independent experiments; ns, non-significant and ****p < 0.0001, all relative to the full control determined by one-way ANOVA with Tukey’s multiple comparison test (**a**). Representative images with a line specifying the perinuclear region (0–4 µm distances from the nucleus) are shown in (**b**). Bar 5 µm.

We then examined the roles of BORCS5-8 and Kinesin-1 in lysosome positioning during starvation-induced autophagy in the BJN-infected BMDMs. To determine this, the location of lysosomes in the BMDMs depleted in BORCS5-8 and Kinesin-1 expressions infected with different mycobacteria induced to undergo autophagy by starvation were analysed by high-content imaging. The total number of lysosomes dispersed across the various sub-cytoplasmic areas was quantified. The illustration from the high-content imaging analysis of Lamp1^+^ lysosomes is shown in Supplementary Fig. S4. The border and nucleus of each infected cell were first determined, and the cytoplasmic areas were then subdivided into 0, 4, 8, 12, 16, and more than 20 μm distance from the nucleus. Subsequently, the number of lysosomes in each subarea was quantitated and the sum of lysosome numbers in each cell was set as 100%. The percentage of perinuclear Lamp1^+^ lysosomes (located between 0 and 4 μm away from the nucleus) and the percentage of peripheral Lamp1^+^ lysosomes (located between 4 μm away from the nucleus and the cell border) were then calculated. In agreement with our previous findings^19^, the increase in lysosome positioning towards the perinuclear region upon autophagy induction by starvation was observed in the scrambled siRNA-transfected H37Rv-infected BMDMs, while such an effect was not seen in the control cells infected with BJN (Fig. 5a,b). On the other hand, the enhanced lysosome positioning towards the perinuclear area could be observed in the BJN-infected BMDMs during starvation-induced autophagy when the expressions of BORCS5-8 and Kinesin-1 were suppressed (Fig. 5a,b). Altogether, these findings indicated that BORCS5-8 and Kinesin-1 play crucial roles in the inhibition of the lysosomal positioning towards the perinuclear region by the BJN resulting in the reduction of lysosomal delivery to the BJN phagosomes and the sparing of the BJN from autophagic restriction during starvation-induced autophagy of host cells.

### BORCS5-8 and Kinesin-1 are not involved in the delivery of mycobacteria to autophagosomes

**Figure 6:**
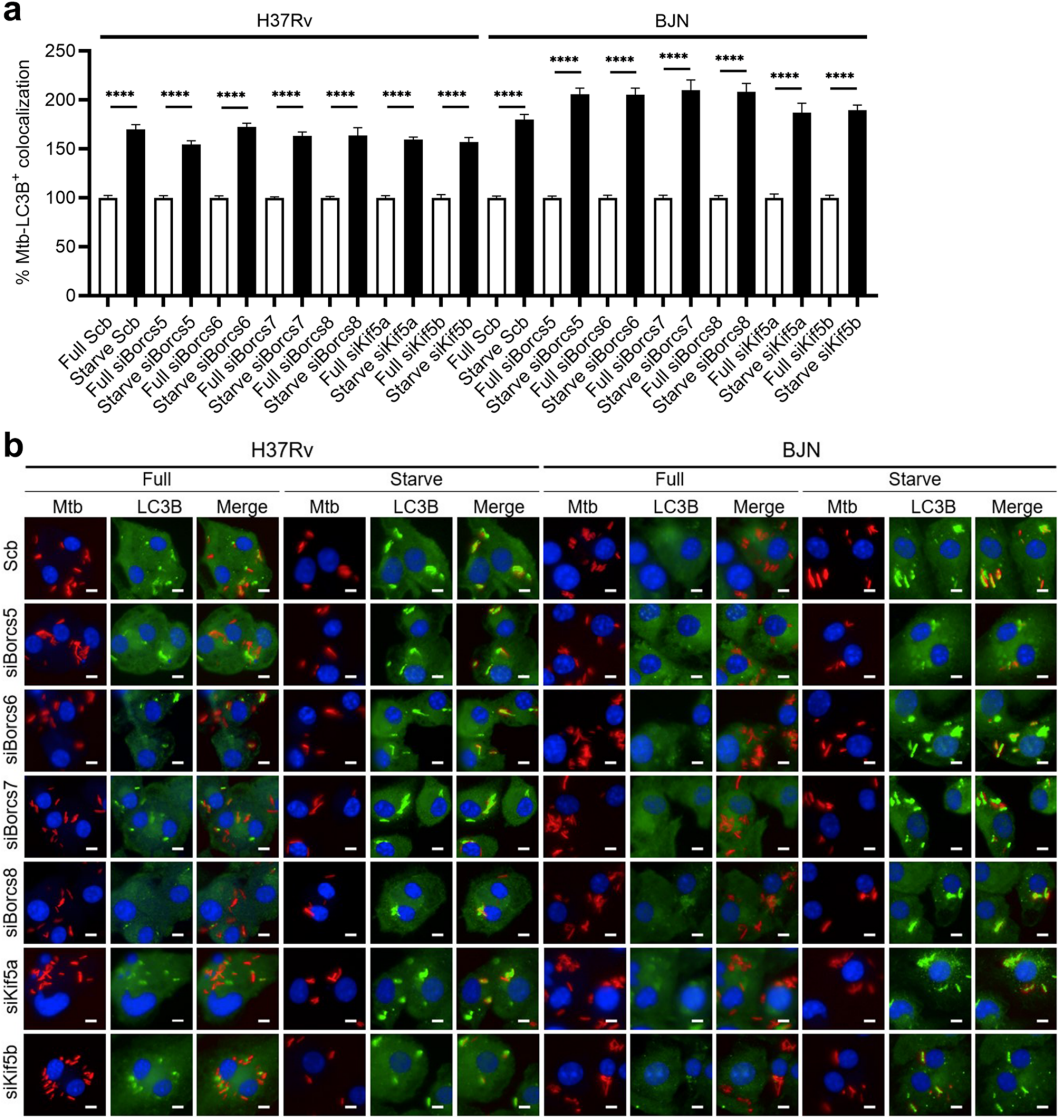
BORCS5-8 and Kinesin-1 are not involved in the BJN delivery to the autophagosomes. (**a,b**) BMDMs deficient in BORCS5-8 and Kinesin-1 expressions were infected with the mCherry-expressing H37Rv or BJN (MOI = 10) for 15 min and chased for 1 h followed by autophagy induction by starvation for 2 h. Cells were fixed and stained with anti-LC3B antibody and Hoechst. Percent mycobacteria-LC3B colocalisation was then analysed by high-content image analysis. Data are means ± SEM from at least three independent experiments; ****p < 0.0001, relative to the full control set of 100% determined by one-way ANOVA with Tukey’s multiple comparison test (**a**). Representative images are displayed in (**b**). Bar 5 µm.

To rule out that the effects of BORCS5-8 and Kinesin-1 on autophagy resistance by the BJN could be due to the roles of these proteins in preventing the delivery of the BJN to autophagosomes, we analysed the colocalisation of different mycobacteria with LC3B, used as the marker for autophagosomes, by high-content imaging during starvation-induced autophagy in BMDMs. As expected, increased colocalisation of LC3B with the H37Rv phagosomes was observed in the scrambled siRNA-transfected BMDMs upon autophagy induction by starvation (Fig. 6a,b). Similar to what was observed in the H37Rv-infected control BMDMs, enhanced BJN-LC3B colocalisation was seen in the scrambled siRNA-transfected BMDMs during starvation-induced autophagy, independent of BORCS5-8 and Kinesin-1 expressions (Fig. 6a,b). Thus, these results indicated that BORCS5-8 and Kinesin-1 do not play any role in the BJN delivery to autophagosomes and confirm their function in enhanced lysosome positioning towards the cell periphery, thereby preventing lysosomal delivery to the BJN phagosomes and thus sparing the BJN from autophagic restriction.

## Discussion

TB is a significant cause of death worldwide. Currently, TB is estimated to account for 1.3 million deaths yearly^1^. Most TB cases occur in Southeast Asia (~ 43%) and the *M. tuberculosis* Beijing genotype is found to be the most prevalent genotype in this region, which is frequently associated with drug resistance^22,33^. Autophagy has been shown to play a crucial role in cellular defence against various pathogens, including *M. tuberculosis*^8,34–36^. Activation of host cell autophagy by starvation increases the delivery of lysosomes to the mycobacterial phagosomes, resulting in their elimination^11,14,16,17^. Although the *M. tuberculosis* reference strain H37Rv could be restricted by autophagy induction, strains belonging to the *M. tuberculosis* Beijing genotype have been reported to have the ability to resist autophagy-mediated elimination in host macrophages^11,19,20^. However, the underlining evasion mechanism by the autophagy-resistant *M. tuberculosis* Beijing strain, dubbed BJN, remains unclear. Our recent work discovered that the BJN could evade autophagic restriction by upregulating the expression of *Kxd1*, a member of the BORC complex, and *Plekhm2*, both of which are involved in lysosome positioning towards the cell periphery in host macrophages, thereby preventing autophagy-mediated lysosomal delivery to its phagosome and elimination^19^. As BORC is a multi-protein complex associated with lysosomes and, together with Arl8, can recruit downstream Kinesin proteins to promote lysosome positioning towards the cell periphery^31,32^, this study further characterised the roles of other BORC complex specific components and Kinesin proteins in autophagy evasion by the BJN.

The current study discovered that the BORC complex specific components, BORCS5-8, and Kinesin-1, but not Kinesin-3, play important roles in evasion from autophagy-mediated restriction by the BJN in host macrophages induced to undergo autophagy by starvation (Figs. 1, 3, Supplementary Fig. S1). Down-regulation of BORCS5-8 and Kinesin-1 could now revert the inhibition of lysosomal delivery to the BJN phagosomes upon autophagy induction by starvation in host cells (Figs. 2, 4). More importantly, depletion of BORCS5-8 and Kinesin-1 enabled the increase in lysosome relocation towards the perinuclear region to be observed in BJN-infected macrophages during starvation-induced autophagy (Fig. 5). Altogether, our findings uncovered new roles for the BORC complex specific components, BORCS5-8, and Kinesin-1 in the autophagy resistance by the *M. tuberculosis* Beijing strain.

The BORC complex consists of eight proteins, in which Kxd1 and BORCS5-8 are the specific components, while BORCS1-3 are shared by the BLOC-1 complex whose function is not involved in lysosome positioning^31,32^. It was revealed that only the knockout and knockdown of the BORC-specific subunits inhibited lysosome positioning towards the cell periphery, resulting in their clustering in the juxtanuclear area^31^. It was also demonstrated that the BORC complex works by one of two different mechanisms, either by recruiting Arl8 followed by Plekhm2 and Kinesin-1 to move lysosomes on the acetylated α-tubulin-enriched microtubules towards the cell periphery or by recruiting Alr8, which directly recruits Kinesin-3 to transport lysosomes on the tyrosinated α-tubulin-enriched microtubes towards the cell periphery^32^. In agreement with our previous work that showed the necessary roles of Kxd1 and Pleckhm2 in the autophagy resistance by the BJN^19^, the current study found that only BORCS5-8 and Kinesin-1, but not Kinesin-3, are important for the BJN’s autophagy evasion, thus confirming the vital role of Plekhm2 in this process. This pathway also provided a drugable target as a recent study identified a Plekhm2-Kinesin-1 inhibitor, Kinesore, from a high-throughput screen^37^. Whether the ability of the BJN to resist autophagic restriction can be inhibited by Kinesore warrants further investigation^19^.

The position of lysosomes in the cytoplasm is known to affect their properties and biological functions^38^. For example, the juxtanuclear lysosomes are more acidic and have higher Cathepsin activity compared to the peripheral lysosomes^39^. The peripheral lysosomes, although having less degradative capacity, play multiple important roles, such as in cell migration^31^, nutrient sensing^40^, cancer cell metastasis^41^, NK cell-mediated cytotoxicity^42^ and mTOR activation^43^. mTOR is a well-known negative regulator of autophagy. As the autophagy-resistant *M. tuberculosis* BJN strain upregulates the expression of a BORC complex specific component, Kxd1, and Plekhm2^19^, and the other BORC complex specific components and Kinesin-1 are shown in this study to be important for the increased lysosome positioning towards the cell periphery during starvation-induced autophagy of host macrophages, it would be interesting to examine whether the BJN can also evade autophagy by activating mTOR. Our investigation, however, showed that there was an increase in BJN-LC3B colocalisation upon autophagy induction by starvation (Fig. 6). In agreement with this data, our previous work also showed that a similar increase in autophagosome formation between H37Rv- and BJN-infected macrophages was observed upon autophagy induction by starvation of host macrophages^11^. Thus, it is unlikely that the BJN evades autophagy by inhibiting autophagosome formation through mTOR activation.

In addition to moving lysosomes, kinesins and dyneins also transport phagosomes bi-directionally along the microtubules^44^. Note that kinesins move organelles towards the microtubule plus-ends while dyneins move organelles towards the microtubule minus-ends. Under basal autophagy condition, the phagosomes are moved bi-directionally on the microtubules with the majority of phagosomes being transported towards the microtubule minus-ends while the remainder of phagosomes being transported towards the microtubule plus-ends^44^. However, upon autophagy induction by starvation, it was shown that the autophagosomes containing the engulfed substrates are moved towards the cell centre in a dynein-dependent manner along the microtubule tracks to fuse with the juxtanuclear acidic lysosomes^40,45^. As our experiments were conducted under autophagy induction condition, not basal autophagy condition, we believe that BORCS5-8 and Kinesin-1 depletion would result in the decreased movement of Mtb-containing autophagosomes towards the microtubule plus-ends, and therefore increasing their maturation by moving them towards the microtubule minus-ends during starvation-induced autophagy. Whether or not our hypothesis is correct awaits future investigation.

Interestingly, a very recent publication demonstrated the important role of the BORC complex in the release of lysosomal cholesterol^46^. Knock out of the BORC complex specific components, Kxd1 and BORCS5-8, in HeLa cells resulted in the accumulation of cholesterol in the lysosomes^46^. Deletion of the BORC complex downstream effectors, Arl8a and Alr8b, and HOPs specific components, VPS39 and VPS41, also increased cholesterol retention in the lysosomes^46^. As *M. tuberculosis* prefers host cell cholesterol as the nutrient source during its growth inside host macrophages, not only for energy^47^, but also for the synthesis of virulence molecules^48^, the BORC complex might play another important role for the autophagy-resistant *M. tuberculosis* BJN strain by increasing the cholesterol level available for mycobacteria. If so, the BJN will be able to not only move the lysosomes towards the cell periphery by upregulating the BORC complex activity in host cells, and thus avoid lysosome fusion to its phagosome and elimination during starvation-induced autophagy, but also activate the egress of lysosomal cholesterol available for its growth and synthesis of virulence molecules. Therefore, whether the BJN upregulates cholesterol release from the host cell lysosome through the BORC complex warrants further investigation.

## Materials and methods

### Cells and bacterial culture

RAW264.7 macrophages (ATCC) were cultivated at 37 °C and 5% CO_2_ in Dulbecco's modified Eagle's medium (DMEM; Gibco) with the addition of 0.37% sodium bicarbonate (Sigma), 10% foetal bovine serum (FBS; Gibco), and 4 mM l-glutamine (Hyclone) (Full medium). BMDMs were extracted from the bone marrow cells of C57/BL6 mice (obtained from Nomura Siam International, Thailand) and grown in an L929 condition media, as previously stated, with modifications^49^. These cells were then frozen in liquid nitrogen until used. All animal procedures were evaluated and approved by the Institutional Animal Care and Use Committee at the Chulalongkorn University Faculty of Medicine (approval protocol number 025/2562) and were performed in compliance with the ARRIVE guidelines**.** All methods were carried out in accordance with relevant guidelines and regulations. The cryopreserved BMDMs were then thawed and grown in DMEM with the addition of 10% FBS (Gibco), 1% HEPES (Gibco), 1% sodium pyruvate (Sigma), 4 mM l-glutamine (Hyclone), and 20% conditioned media from L929 cell (Full medium). Earle’s Balanced Salt Solution (EBSS; Gibco) (starvation medium) was used for autophagy induction. Middlebrook 7H9 medium with the addition of 10% oleic acid–albumin–dextrose–catalase (OADC; BD), 0.05% Tween 80, 0.2% glycerol, hygromycin (100 g/mL; Invitrogen) was used to cultivate the mCherry-expressing *M. tuberculosis* reference strain H37Rv and autophagy-resistant BJN strain^19,20^ at 37 °C. Before the research started, the mycobacterial cultures in the log phase were collected, washed twice with PBS, resuspended in a complete medium, homogenised to create single-cell mycobacteria, and then measured for absorbance at 600 nm.

### Fluorescent dyes, antibodies and siRNAs

Anti-Lamp1 monoclonal antibody (DSHB) was diluted at 1:25, anti-Cathepsin D polyclonal antibodies (R&D Systems) were diluted at 1:50, and anti-LC3 polyclonal antibodies (MBL) were diluted at 1:200 for immunofluorescence assay. In addition, nuclear staining was performed using a 1:500 dilution of Hoechst 33342 (Thermo Fisher Scientific). All secondary antibodies (Thermo Fisher Scientific) were diluted at 1:400. Scrambled control siRNAs and all siRNAs used in this study were from Dharmacon.

### Downregulation of RNA expression

The siRNA-mediated gene knockdown was carried out as described previously^50^. Briefly, Raw264.7 macrophages and BMDMs were resuspended in 90 µL of solution V (for Raw264.7 cells; Lonza) or solution for mouse macrophages (for BMDMs; Lonza), respectively. The cell suspension was mixed with either 1.5 μg of scrambled siRNAs or siRNAs against *Borcs5*, *Borcs6*, *Borcs7*, *Borcs8*, *Kif5a*, *Kif5b* and Kif1b, and then transferred to a nucleofection cuvette and nucleofected using the Amaxa Nucleofector device (Amaxa Biosystems) set to either programme D-032 (for RAW264.7 macrophages) or Y-001 (for BMDMs). At 24 h post-transfection, the cells were collected and plated in preparation for the experiments.

### RNA extraction and qRT-PCR analysis

Trizol was used to isolate total RNAs at 48 h post siRNA transfection. Briefly, the medium was withdrawn, and 1 mL of Trizol (Thermo Fisher Scientific) was added to the cells. After 5 min incubation at room temperature, nucleic acids were extracted with phenol/chloroform solution and precipitated with isopropanol. Next, DNaseI (Thermo Fisher Scientific) was introduced to break down the genomic DNAs. An RNeasy kit (Qiagen) was then used to isolate the total RNAs. A NanoDrop spectrophotometer (Denovix) was used to measure RNA concentration. Finally, reverse transcription with random hexamers (Promega) was performed on total RNAs (500 ng). The corresponding gene-specific primers were obtained commercially (Ward Medic; Supplementary Table S1). The synthesized cDNAs were used as templates in qRT-PCR experiments using a thermocycler (Rotor-Gene Q, Qiagen) with reactions comprising 0.1 mM forward and reverse primers, HotStarTaq DNA buffer and polymerase (Qiagen), 10 mM dNTPs (Promega), 4 mM MgCl2 (Qiagen), and SYBR green (Invitrogen). Negative controls were implemented by the use of reaction-minus templates. Rotor-Gene Q series software version 2.3.5 was used to analyse the signals. Evaluation of the melting curves to guarantee the specificity of the PCR products was conducted, and it was determined that the threshold signals were more than 95% effective. The endogenous *Gapdh* transcript was used to normalise the levels of the gene signals. Using the 2^−∆∆Ct^ approach, the results were given as relative quantifications.

### Mycobacterial infection and survival assay

Raw264.7 macrophages were grown to 80% confluence in 75 cm^2^ tissue culture flasks, while BMDMs were grown in non-treated Petri dishes. After collecting the cells, they were seeded onto 96-well black plates and given 16–18 h to rest. The cells were then infected with mCherry-expressing H37Rv or BJN at an MOI of 10. After four PBS washes, the cells were treated with EBSS to induce autophagy by starvation for 4 h. Next, the cells were fixed with 4% paraformaldehyde and the nucleus was stained for 15 min with Hoechst. High-content imaging system (Operetta, PerkinElmer) was then used to obtain confocal images (14 fields per well) and determine the fluorescent signals of each cell at × 40 magnification. The number of intracellular mycobacteria per cell was automatically calculated using the Columbus image analysis server (PerkinElmer, USA). Percent mycobacterial survival was then computed and compared across various conditions.

### Lysosome distribution and colocalisation with mycobacteria

Mycobacteria colocalisation with lysosome and autophagosome markers was determined by immunofluorescence assay followed by high-content image analysis, as described previously^19,20,50,51^. In brief, host macrophages (2.5 × 10^4^ cells per well) were plated onto 96-well black plates. At 48 h after siRNA transfection, cells were infected with mCherry-expressing H37Rv or BJN at an MOI of 10 for 15 min, washed with complete media four times to get rid of the uninternalised mycobacteria, and then chased for 1 h. Autophagy was then induced by washing the cells with PBS four times and treating the cells with EBSS for 2 h. After that, cells were fixed with 4% paraformaldehyde and stained with anti-Cathepsin D (for RAW264.7 macrophages) or anti-Lamp1 and anti-LC3 (for BMDMs), followed by staining with Alexa488 conjugated secondary antibodies. Finally, the nucleus was stained for 15 min with Hoechst. The confocal images (14 fields per well) and fluorescent signals of each cell were acquired by high-content imaging system (Operetta, PerkinElmer) at × 40 magnification. Percent mycobacteria-marker colocalisation was then automatically quantified by the Columbus image analysis server (PerkinElmer, USA).

The lysosome distribution in mycobacteria-infected BMDMs was automatically determined by the Columbus image analysis server (PerkinElmer, USA), as described above, but analysed for the number of Lamp1^+^ lysosomes in each cytoplasmic subregion in the infected cells. To do this, the cytoplasmic area was first defined by the distance between the nucleus and plasma membrane boundary. Then, the cytoplasmic area was subdivided into subareas of 0, 4, 8, 12, 16, and more than 20 μm distance from the nucleus. Percent perinuclear Lamp1^+^ lysosomes (located between 0 and 4 μm distance from the nucleus) and percent peripheral Lamp1^+^ lysosomes (located between 4 μm distance from the nucleus and the cell boundary) were then automatically determined and compared between conditions by the Columbus image analysis server (PerkinElmer, USA).

### Statistical analysis

Unless otherwise specified, experiments were conducted at least three times. The data were pooled and the mean ± standard error of the mean (S.E.M.) was determined. Prism (GraphPad) software was then used to conduct the statistical analysis. A p-value of less than 0.05 was considered statistically significant.

## Supplementary Information

Supplementary Information.

## Data Availability

The datasets generated during the current study are available from the corresponding author upon reasonable request.
