# Supplementary material for: BORC complex specific components and Kinesin-1 mediate autophagy evasion by the autophagy-resistant Mycobacterium tuberculosis Beijing strain
Source: Sci Rep. 2023 Jan 30;13:1663. doi: 10.1038/s41598-023-28983-5 (PMC9886903; doi:10.1038/s41598-023-28983-5)
Supplement: Supplementary file 1 — Supplementary Information. [file 41598_2023_28983_MOESM1_ESM.pdf]

Supplementary Information

BORC complex specific components and Kinesin-1 mediate autophagy evasion by the autophagy-resistant *Mycobacterium tuberculosis* Beijing strain

Janpen Tunganuntarat[1], Phongthon Kanjanasirirat[2], Tanawadee Khumpanied[2], Salisa Benjaskulluecha[3,4], Benjawan Wongprom[4], Tanapat Palaga[3,4], Tegar Adriansyah Putra Siregar[1,5], Suparerk Borwornpinyo[2,6], Angkana Chaiprasert[7,8], Prasit Palittapongarnpim[1,9,10] & Marisa Ponpuak[1,10*]

Supplementary Figure

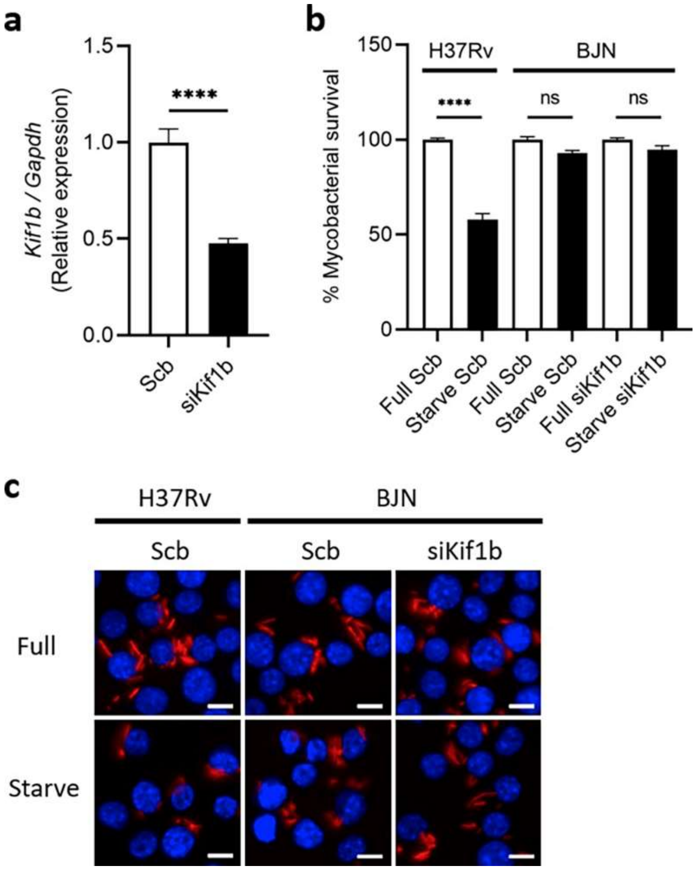

Supplementary Figure S1. Kinesin 3 is not important for autophagy resistance by the BJN. (a) RAW264.7 macrophages were transfected with scrambled control siRNAs or siRNAs against *Kif1b*, which encodes Kinesin 3. At 48 hr after transfection, *Kif1b* expression levels were determined by qRT-PCR. Data are means ± SEM from at least three independent experiments; ****$p < 0.0001$ was determined by a two-tailed unpaired Student's t-test. **(b, c)** Kif1b-deficient or control RAW264.7 macrophages were infected with mCherry-expressing H37Rv or BJN for 1 hr and induced to undergo autophagy by starvation for 4 hr. Cells were then fixed and processed for high-content imaging. The number of intracellular mycobacteria per cell was then determined and per cent mycobacterial survival was calculated and compared. Data are means ± SEM from at least three independent experiments; ns, non-significant and ****$p < 0.0001$, relative to the full control set of 100% were determined by one-way ANOVA with Tukey's multiple comparison test **(b)**. Representative images are displayed in **(c)**. Bar 10 μm.

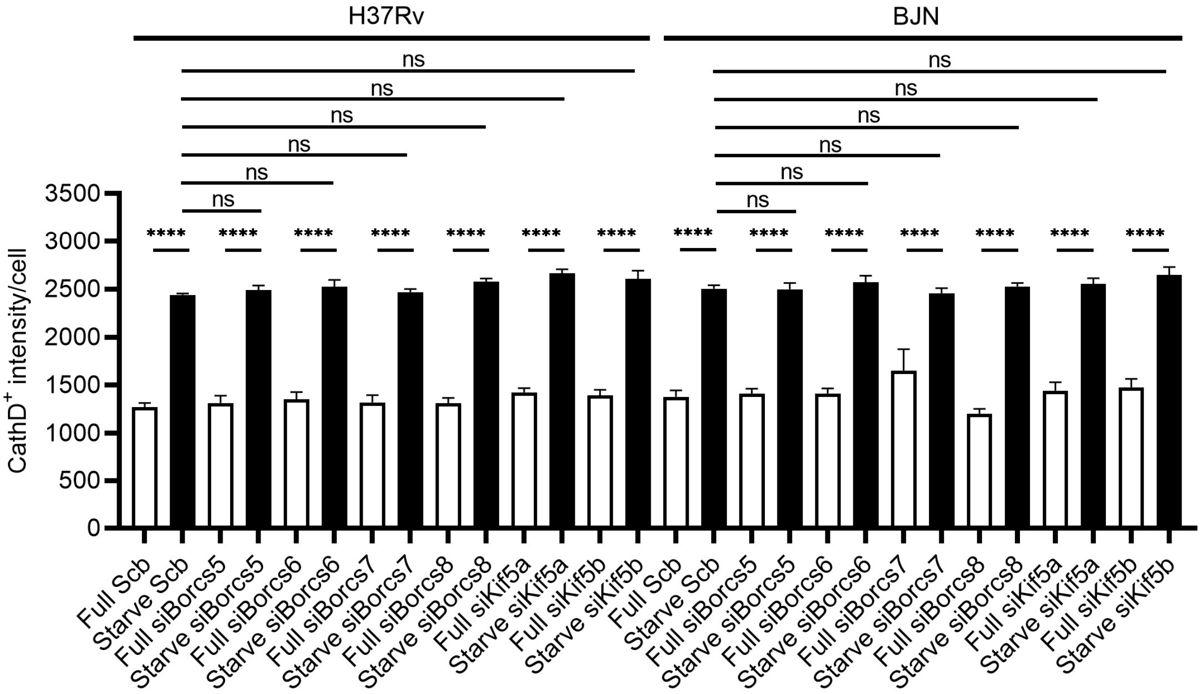

Supplementary Figure S2. The fluorescence intensity of Cathepsin D is increased during starvation. BORCS5-8- and Kinesin-1-depleted RAW264.7 macrophages were infected with the mCherry-expressing H37Rv or BJN for 15 min and chased for 1 hr. Autophagy was then induced by starvation for 2 hr. Cells were fixed and stained for lysosomes using an anti-Cathepsin D antibody followed by nuclear labelling with Hoechst. Cathepsin D intensity per cell was then analysed by high-content image analysis. Data are means ± SEM from at least three independent experiments; ns, non-significant and ****$p < 0.0001$, all relative to the full control determined by one-way ANOVA with Tukey's multiple comparison test.

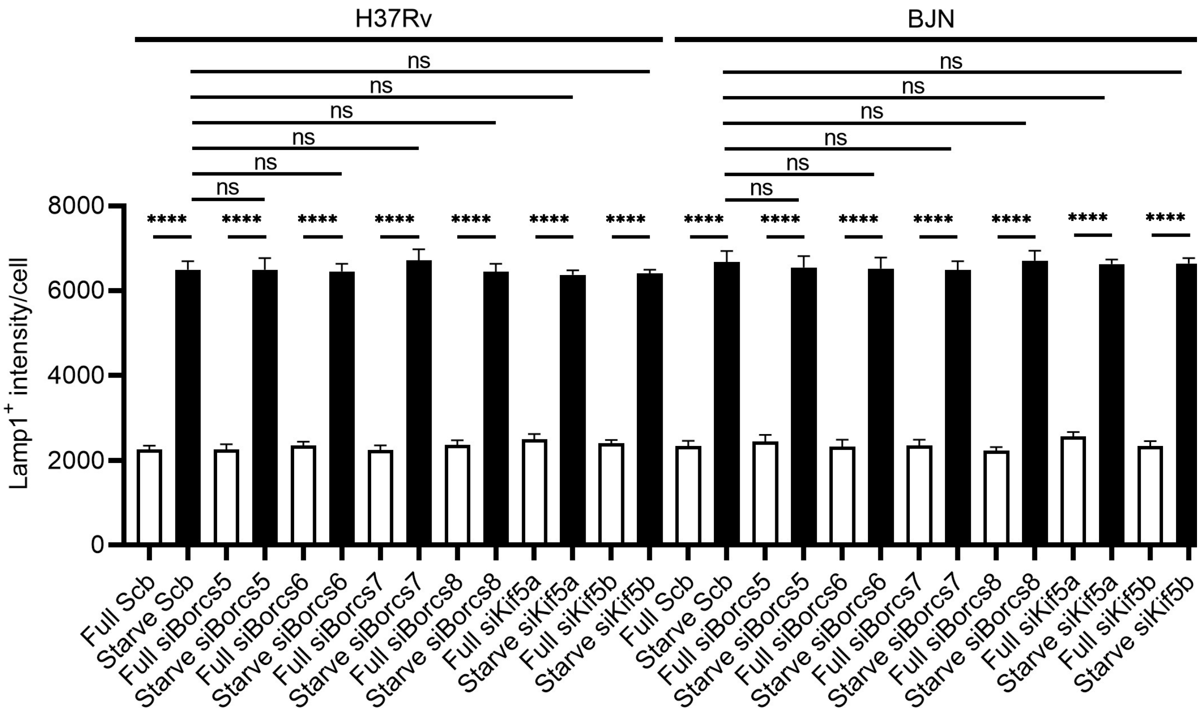

Supplementary Figure S3. Lamp1 fluorescence intensity is augmented during starvation. BMDMs deficient in BORCS5-8 and Kinesin-1 were infected with the mCherry-expressing H37Rv or BJN for 15 min and chased for 1 hr. Autophagy was then induced by starvation for 2 hr. Cells were fixed and stained for lysosomes using an anti-Lamp1 antibody followed by nuclear staining with Hoechst. Lamp1 intensity per cell was then analysed by high-content image analysis. Data are means ± SEM from at least three independent experiments; ns, non-significant and ****$p < 0.0001$, all relative to the full control determined by one-way ANOVA with Tukey's multiple comparison test.

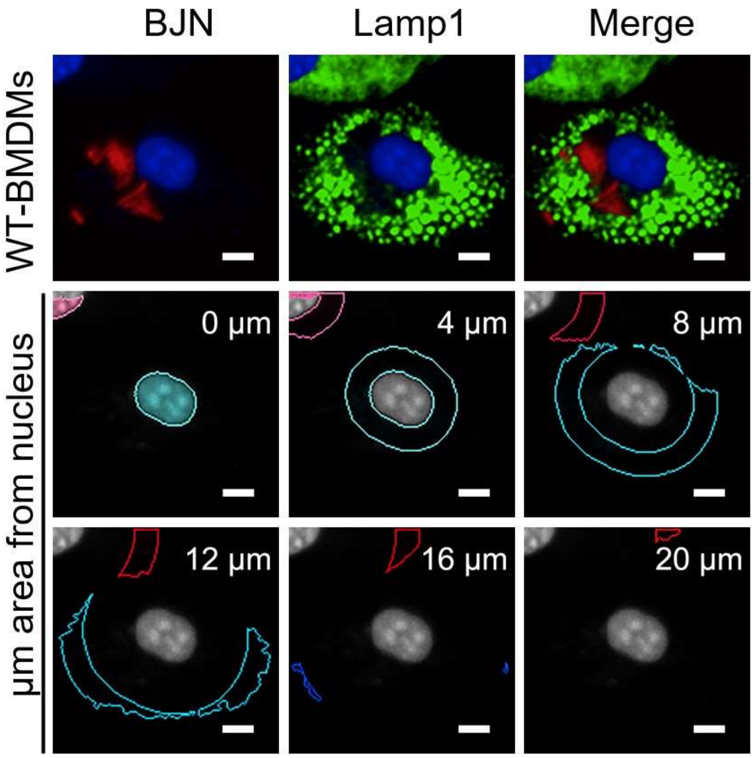

Supplementary Figure S4. The quantification of Lamp1+ lysosomes in the cells. BMDMs were infected with the mCherry-expressing mycobacteria for 15 min and chased for 1 hr. Autophagy was then induced by starvation for 24 hr. Cells were stained with anti-Lamp1 antibody and Hoechst and then processed for high-content image analysis. The number of Lamp1^{+} lysosomes in each cytoplasmic subregion of the mycobacteria-infected BMDMs was quantified. Representative images with a line specifying the regions at various distances from the nucleus are shown. The percentage of perinuclear Lamp1^{+} lysosomes (0-4 μm distance from the nucleus) and peripheral Lamp1^{+} lysosomes (4 μm from the nucleus and cell boundary) were then calculated and compared in each cell as shown in Figure 5a. Bar 5 μm.

Supplementary Table

Supplementary Table S1. Primer sequences used for qRT-PCR in this study.

Target gene	Orientation	Primer sequence (5′–3′)	Annealing temperature (°C)
Borcs5	Forward	5'-GGAGCGCCAGAAAAGATACG-3'	55
	Reverse	5'-CGTCTGGTCTATGCCCATCT-3'	
Borcs6	Forward	5'-AGTGACCAGGAGCATAGAGG-3'	55
	Reverse	5'-TCTAACGTACAGGCATCCCC-3'	
Borcs7	Forward	5'-GGATCACTTGTGCCCAGAAAG-3'	55
	Reverse	5'-GCCATTATCCTTGCTCCGTG-3'	
Borcs8	Forward	5'-GTCTATGTCCTTGCCAACGAGC-3'	55
	Reverse	5'-TACTCCACGGTGTATATGGCGC-3'	
Kif5a	Forward	5'-GTGCTGAATGGGCTGATGAG-3'	55
	Reverse	5'-GACATCGCTTAACCACGGAC-3'	
Kif5b	Forward	5'-GCGAGATGAAGTGGAGGCAAAG-3'	55
	Reverse	5'-CTCTTGGTCTGTAGCCTTCAGC-3'	
Kif1b	Forward	5'-CAGAGCGGGATCAGTGGTAT-3'	55
	Reverse	5'-ACTCCACCTGTGCTGTAGAC-3'	
Gapdh	Forward	5'-GTTGTCTCCTGCGACTTCA-3'	55
	Reverse	5'-GGTGGTCCAGGGTTTCTTA-3'	
